# Three-dimensional CT texture analysis of anatomic liver segments can differentiate between low-grade and high-grade fibrosis

**DOI:** 10.1186/s12880-020-00508-w

**Published:** 2020-09-21

**Authors:** Bettina Katalin Budai, Ambrus Tóth, Petra Borsos, Veronica Grace Frank, Sonaz Shariati, Bence Fejér, Anikó Folhoffer, Ferenc Szalay, Viktor Bérczi, Pál Novák Kaposi

**Affiliations:** 1 Department of Radiology, Medical Imaging Centre, Semmelweis University Faculty of Medicine, Korányi Sándor street 2., Budapest, H-1083 Hungary; 2 1st Department of Internal Medicine, Semmelweis University Faculty of Medicine, Korányi Sándor street 2/a, Budapest, H-1083 Hungary

**Keywords:** Machine learning, Texture analysis, Liver fibrosis, Computed tomography, Prediction model, Radiomics

## Abstract

**Background:**

CT texture analysis (CTTA) has been successfully used to assess tissue heterogeneity in multiple diseases. The purpose of this work is to demonstrate the value of three-dimensional CTTA in the evaluation of diffuse liver disease. We aimed to develop CTTA based prediction models, which can be used for staging of fibrosis in different anatomic liver segments irrespective of variations in scanning parameters.

**Methods:**

We retrospectively collected CT scans of thirty-two chronic hepatitis patients with liver fibrosis. The CT examinations were performed on either a 16- or a 64-slice scanner. Altogether 354 anatomic liver segments were manually highlighted on portal venous phase images, and 1117 three-dimensional texture parameters were calculated from each segment. The segments were divided between groups of low-grade and high-grade fibrosis using shear-wave elastography. The highly-correlated features (Pearson r > 0.95) were filtered out, and the remaining 453 features were normalized and used in a classification with k-means and hierarchical cluster analysis. The segments were split between the train and test sets in equal proportion (analysis I) or based on the scanner type (analysis II) into 64-slice train 16-slice validation cohorts for machine learning classification, and a subset of highly prognostic features was selected with recursive feature elimination.

**Results:**

A classification with k-means and hierarchical cluster analysis divided segments into four main clusters. The average CT density was significantly higher in cluster-4 (110 HU ± SD = 10.1HU) compared to the other clusters (c1: 96.1 HU ± SD = 11.3HU; *p* < 0.0001; c2: 90.8 HU ± SD = 16.8HU; p < 0.0001; c3: 93.1 HU ± SD = 17.5HU; p < 0.0001); but there was no difference in liver stiffness or scanner type among the clusters. The optimized random forest classifier was able to distinguish between low-grade and high-grade fibrosis with excellent cross-validated accuracy in both the first and second analysis (AUC = 0.90, CI = 0.85–0.95 vs. AUC = 0.88, CI = 0.84–0.91). The final support vector machine model achieved an excellent prediction rate in the second analysis (AUC = 0.91, CI = 0.88–0.94) and an acceptable prediction rate in the first analysis (AUC = 0.76, CI = 0.67–0.84).

**Conclusions:**

In conclusion, CTTA-based models can be successfully applied to differentiate high-grade from low-grade fibrosis irrespective of the imaging platform. Thus, CTTA may be useful in the non-invasive prognostication of patients with chronic liver disease.

## Background

Hepatic fibrosis can result from various types of chronic damaging factors, including viral infections, toxins, metabolic diseases, alcoholic or non-alcoholic steatohepatitis, autoimmune disorders, and chronic biliary diseases, among others. Patients with advanced-stage fibrosis and cirrhosis are at increased risk of developing portal hypertension, hepatic insufficiency, and hepatocellular carcinoma. Therefore, early detection and staging of liver fibrosis have great clinical importance.

Percutaneous liver biopsy is the current gold standard method for staging fibrosis. It is a highly invasive, painful procedure with considerable sampling variability and potential of complications. In recent guidelines on the treatment of chronic viral hepatitis, non-invasive methods are recommended for the initial assessment of fibrosis. Meanwhile, liver biopsy is reserved for cases where there is uncertainty or potential additional etiologies [1–3]. Non-invasive methods have emerged in recent years, including ultrasound-based and MRI-based elastography techniques, which offer a promising new paradigm for diagnosing and staging fibrosis [3, 4]. CT has been frequently used for follow-up patients with hepatic fibrosis and to identify liver malignancies. Although classic signs of liver cirrhosis are well recognizable on CT scans, conventional CT techniques have low sensitivity for quantifying the more subtle architectural changes of the parenchyma caused by fibrosis.

CT-texture analysis (CTTA) is one of the most developing areas of radiomics that can quantitatively describe the heterogeneity and the distribution of pixel or voxel grey-levels on CT scans. CTTA builds on complex quantitative imaging features invisible to the human eye and that are constructed by various mathematical transformations of the original image. CTTA has been used with success for objective and quantitative assessment of tissue heterogeneity in benign and malignant lesions in many different organs, including breast, lung, thyroid, and liver [5]. Previous studies have already achieved significant success with radiomic analysis of CT images in liver fibrosis. However, these methods were tested on **a** single cross-section of the liver [6, 7], or smaller regions of interest [8, 9], and they did not assess cirrhosis related changes in all three dimensions of the liver volume. Previous reports did not analyze the effect of variable scanning parameters such as the dynamic of contrast enhancement and differences between scanners during CTTA based characterization of liver fibrosis.

This work aims to demonstrate the value of three-dimensional CTTA in the evaluation of diffuse liver disease. This paper attempts to identify the source of variance in large scale texture datasets extracted from volumes of fibrotic liver parenchyma. Finally, we aimed to develop CTTA based prediction models, which can be used for staging fibrosis in different anatomic liver segments irrespective of variations in scanning parameters.

## Methods

### Study population

The institutional ethics committee of our university has approved the present study according to the World Medical Association guidelines and Declaration of Helsinki, revised in 2000 in Edinburgh. As this is a retrospective case-control study, the need for written patient consent was waived by the ethics committee; in compliance with our institutional protocols, written informed consent was obtained before the CT and ultrasound scans from all patients. The CT scans of sixteen female (ages 22–72, mean age 52 years) and sixteen male patients (ages 42–75, mean age 63 years) with chronic liver diseases who had both an abdominal CT scan and a point shear wave elastography (pSWE) measurement at our institution between September 2016 and January 2019 were retrospectively selected. Two patients were excluded from the study due to a lack of contrast-enhanced CT. The shear wave elastography measurement was performed within six months of the CT scan. All patients in this study had been clinically diagnosed and followed for chronic liver disease due to various etiologies (Table 1).

**Table 1:** Distribution of demographics, etiology, and fibrosis stage in the patient cohort

Patients	Number	Age (range)
Female	16	52 years (22–72)
Male	16	63 years (42–75)
**Etiology of liver fibrosis**		**Ratio (%)**
chronic HCV	14	43.8% (14/32)
toxic hepatitis	7	21.9% (7/32)
PBC, PSC, AIH	3	9.4% (3/32)
chronic HBV	1	3.1% (1/32)
Unknown	7	21.9% (7/32)
**Fibrosis stage**		
Low-grade (< 9.5 kPa)	11	34.4% (12/32)
High-grade (≥9.5 kPa)	21	65.6% (20/32)

The liver pSWE was completed with the S-Shearwave™ application and an RS85 Prestige ultrasound scanner (Samsung Medison, Hongcheon, Korea) as part of the patients’ regular follow-up. Patients were divided into two groups according to their pSWE measurements: low-grade fibrosis including F0, F1, and F2 METAVIR stages (11 patients, pSWE< 9.5 kPa), and high-grade fibrosis including F3 and F4 METAVIR stages (21 patients, pSWE≥9.5 kPa) as described previously [10].

### CT examination and texture analysis

The patients were examined according to our routine diagnostic protocols on either a 16-slice Brilliance or a 64-slice Ingenuity Core 64 CT scanner (Philips Healthcare, Best, the Netherlands). The scanners had the following settings: tube voltage of 120 kV; automatic tube current modulation in the range of 193-458mAs; rotation time 0.5 s, collimation 16 × 1.5 mm, or 64 × 0.625 mm, spiral pitch 0.813 or 0.798, pixel spacing 0.916 or 0.695 mm for the 16 and 64-slice scans respectively. The 16-slice acquisitions were routinely reconstructed with filtered back projection, and 64-slice scans with the iDose4™ hybrid iterative reconstruction kernel. A non-ionic, iodinated contrast agent (range of concentration: 350-370 mg/ml) was administered intravenously using a power injector with an injection rate of 2–3.5 ml/sec. The amount adjusted to body weight (0.5 g iodine/kg). The injection rate was adjusted to achieve a fixed injection time of 30 s. The bolus tracking method was used for timing the scan, where a region of interest (ROI) was placed in the lumen of the descending aorta above the diaphragm. The portal venous (PV) phase scan was initiated 60 s after the aortic enhancement in the ROI exceeded the 150 Hounsfield Unit (HU) threshold.

**Fig. 1:**
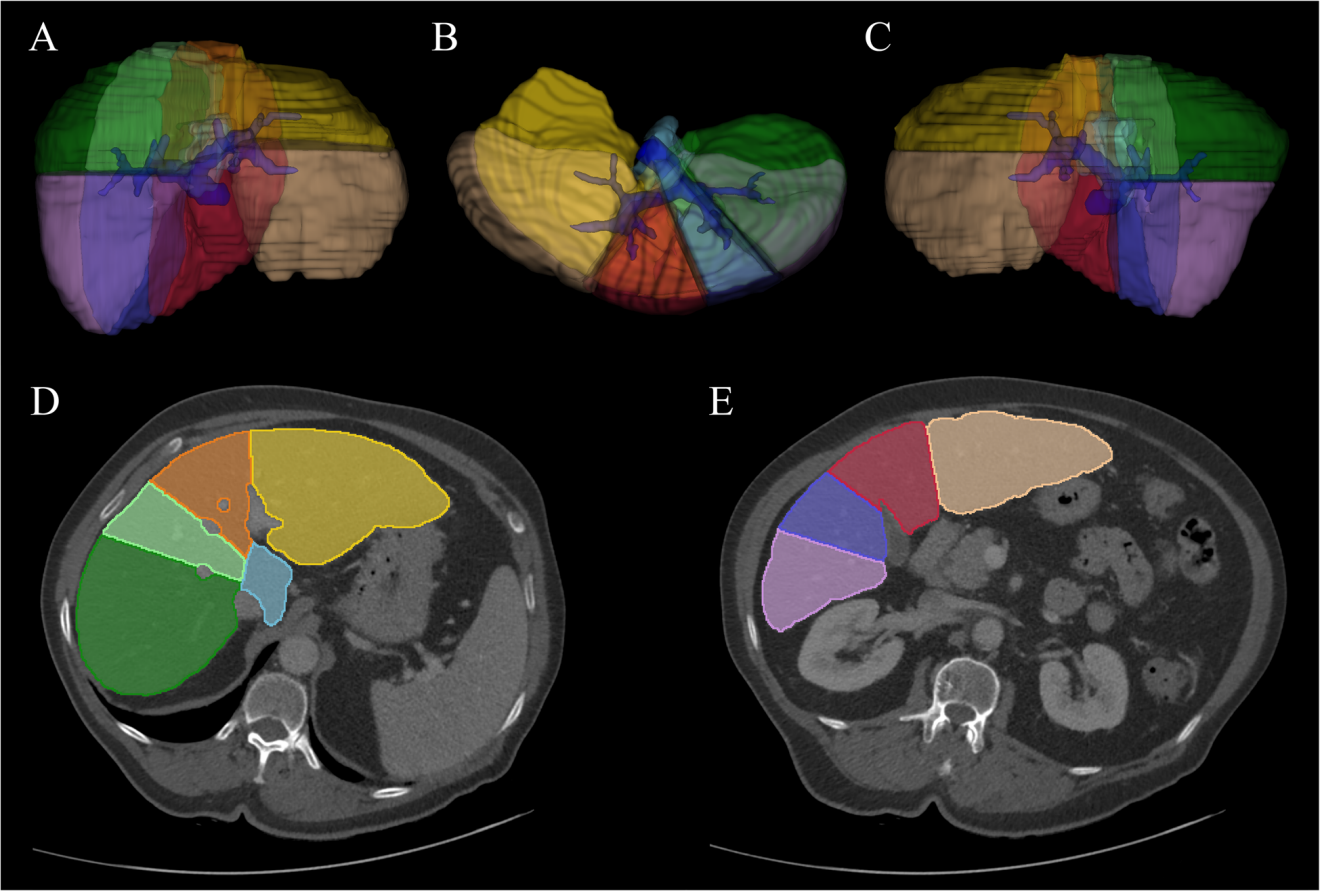
The anatomic liver segments were manually segmented for the three-dimensional texture analysis. **a** is the anterior, **b** is the inferior and **c** is the posterior view of the segmented liver on a three-dimensional volume reconstruction. **d** The contrast-enhanced portal venous phase axial images of a cirrhotic liver after manual segmentation show the upper II., IV.a, VII., VIII. and (**e**) the lower I., III., IV.b, V., VI., Couinaud segments

The PV phase series were reconstructed to 5 mm slice thickness with no interslice gap. The anonymized images were transferred in DICOM format for segmentation and feature extraction with the 3DSlicer 4.8.1 software (www.slicer.org) [11]. Whole liver maps and right and left lobes, and the eight anatomical liver segments, were manually labeled in consecutive slices covering the entire liver volume (Fig. 1). The axial slice with the greatest cross-section at the bifurcation of the portal vein was used to separate upper and lower segments in each lobe, which provided a good approximation of the anatomical liver segments.

In general, twelve liver segments, including nine anatomic liver segments, right and left lobes, and the whole liver were manually annotated in 30 patients on PV scans. One of the patients had prior resection of the right posterior-lateral segment (S6), we also detected circumscribed lesions such as hepatic cysts in the S3 segment of one and in the S4A and S4B segments of two patients. Therefore, altogether six liver segments were omitted from the analysis and the final dataset consisted of 354 liver segments.

Altogether, 1117 texture parameters (TP) were calculated for each segment using the PyRadiomics package [12, 11]. From the original images, 18 first-order intensity features, 13 shape-based features, 23 grey level co-occurrence matrix features (GLCM), 16 grey level run-length matrix features (GLRLM), 16 grey level size zone matrix features (GLSZM), 14 grey level dependence matrix features (GLDM), and five neighboring grey-tone difference matrix features (NGTDM) were extracted. After the original three-dimensional images were transformed with wavelet and Laplacian of Gaussian (LoG) filters, 276 features were calculated with LoG kernel sizes of 3 mm, 6 mm, and 9 mm, and 736 features after discrete wavelet transformations. The average CT density was measured in HU in all anatomic segments in the portal venous phase using circular ROIs. The texture analysis data are available in Additional file 1 and Additional file 2.

### Analysis of the texture dataset

The TPs were log-transformed, and a correlation matrix was calculated using the Pearson correlation. Highly correlated features with an *r* coefficient > 95% were discarded. The remaining 453 parameters were median centered and scaled to the interquartile range.

A *K*-means clustering was used for the unsupervised classification of the liver segments. The optimal number of *k* was determined with silhouette analysis [13]. We compared the continuous variables among the *k*-mean clusters with ANOVA and post hoc Tukey’s tests. Contingency tables were calculated for the categorical variables, and the F-test was used to determine significance in the pairwise comparisons. A hierarchical cluster analysis was performed on each of the *k*-means clusters. The Pearson correlation was used to calculate the distance matrix, the dendrograms were built with Ward. D linkage, and heatmaps were drawn to visualize the features.

We classified liver segments as either low-grade or high-grade fibrosis based on a 9.5 kPa SWE cutoff. We performed a receiver-operating characteristics (ROC) analysis with each of the texture parameters. The area under the curve (AUC) was used as a performance metric of the features. We calculated the AUC estimates and the influence curve based on 95% confidence intervals using 5-fold cross-validation [14]. A univariate linear regression model was constructed to identify predictors of liver stiffness. We used bootstrapping with 1000 replications to calculate the 95% confidence intervals of the model coefficients. A principal component analysis showed that the first 45 components explain 95% of the total variance of the dataset. Therefore, *p*-values from multiple comparisons were not corrected with the number of test hypotheses as in a Bonferroni correction, rather a *p* < 0.0011 (0.05/45) cutoff was used to determine statistical significance [15].

**Fig. 2:**
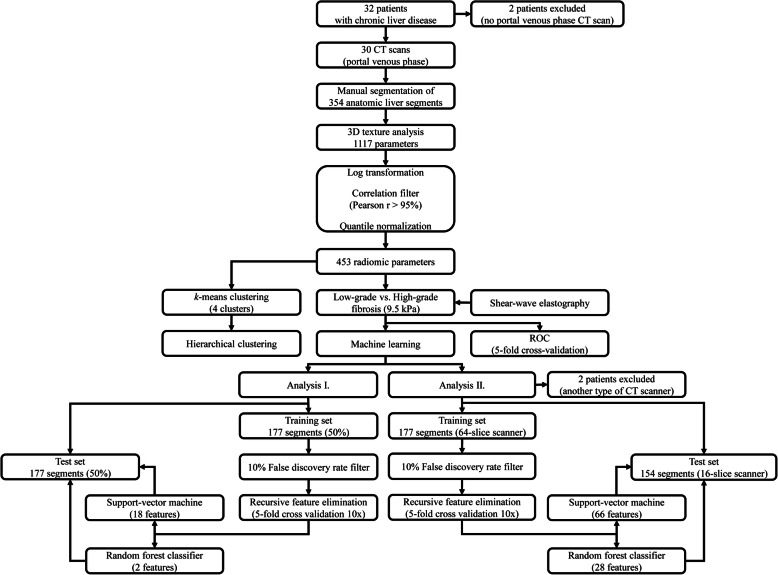
Flow chart shows the steps of data analysis. We manually highlighted anatomic liver segments on portal venous phase CT scans of patients with chronic liver diseases. A three-dimensional texture analysis generated 1117 features out of each segment. The highly correlated features were filtered out from the dataset before normalization to the interquartile range. An unsupervised *k*-means and hierarchical clustering were performed with all segments. The univariate classification rate of the features for low-grade vs. high-grade fibrosis was tested in a receiver operating curve analysis. The cutoff at 9.5 kPa of shear-wave elastography was used as a reference. A machine learning pipeline was used to build models that could predict high-grade vs. low-grade fibrosis based on selected texture features. In the first analysis, the segments were randomly split between equal size train and test sets. In the second analysis, the segments scanned with a 64-slice scanner constituted the train set, and segments scanned with a 16-slice scanner were assigned to the test set. The models were optimized and validated on the corresponding training and the test sets, respectively

Four machine learning models were constructed for the supervised classification of low-grade and high-grade fibrosis. In the first analysis, the samples were split between the train and test sets in equal proportions. In a second analysis, the cases were split between the train and test sets based on the scanner types. Features with more than 10% univariate false discovery rate (FDR) were filtered out in the training set. We chose random forest (RFC) and the support vector machine (SVM) classifiers for the models. A grid-search pipeline was used to determine the optimal hyperparameters of the models. We used recursive feature elimination (RFE) to calculate the optimal number of features in the classifier, rank features by relevance, and then fit the model on the training set. Ten repeats of a stratified 5-fold cross-validation were performed at each step. The final model’s performance was also validated on the independent test set (Fig. 2). The categorical variables are reported as ratio and percentage, the continuous variables as mean and standard deviation (SD). The AUC scores are reported as median and 95% confidence interval (CI). We used the R × 64 3.5.3 software (www.r-project.org) for data analysis.

## Results

### Unsupervised classification of liver segments

**Fig. 3:**
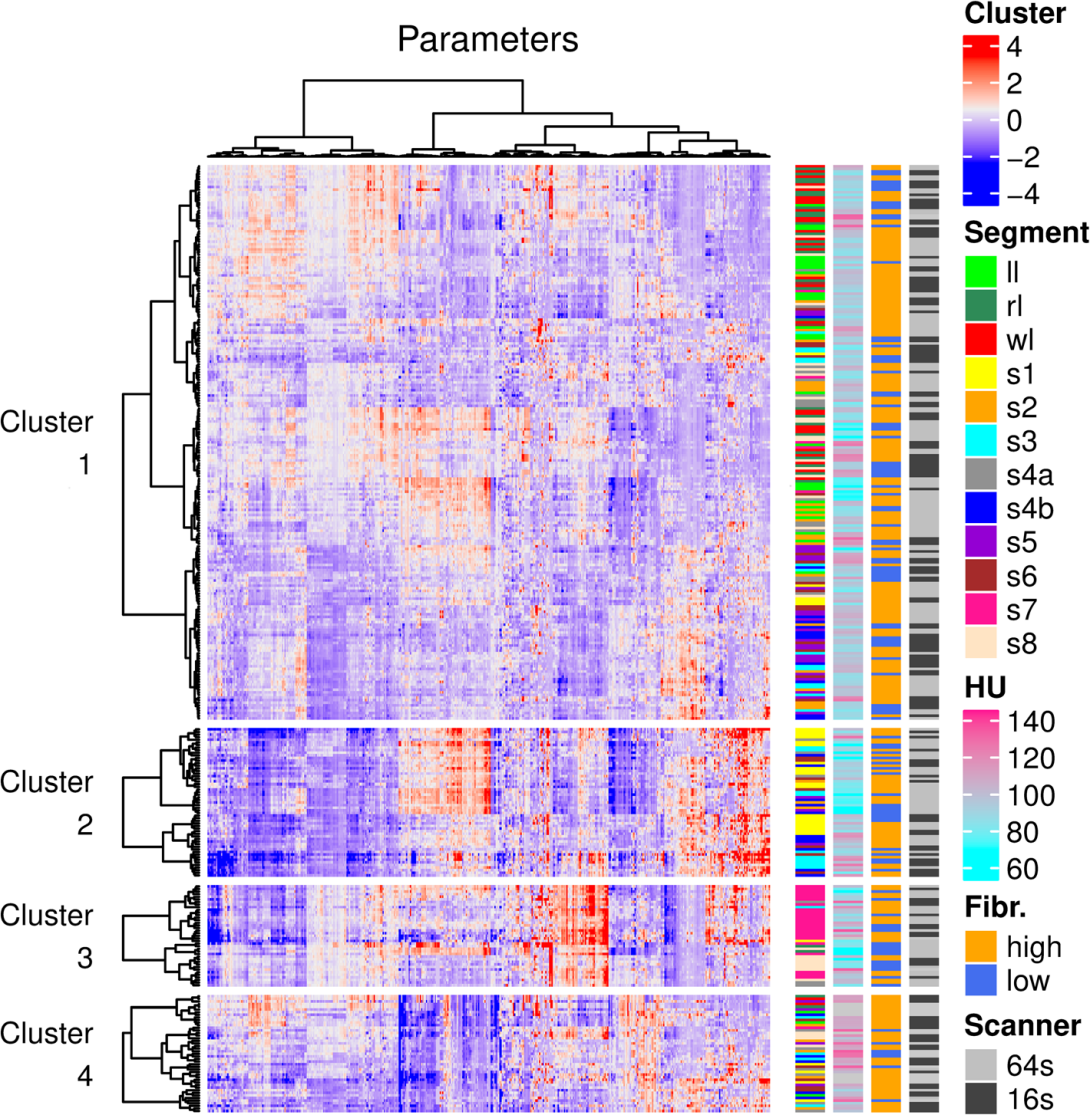
*K-*means and hierarchical cluster analysis of liver segments. Liver segments were grouped by *k*-means clustering into four clusters. The optimal number of clusters was determined with the silhouette method. The heatmap was constructed by hierarchical clustering of liver segments (rows) and texture parameters (columns) in each of the four *k*-means clusters. For drawing the dendrograms, the Pearson correlation was used as a distance metric, and linkage was determined by the Ward. D method. The distribution of clinical and technical variables was also compared among the clusters (color bars)

The texture analysis extracted 1117 parameters from the 354 anatomic liver segments, which were manually segmented on the portal venous phase CT scans of 30 patients. After the highly correlated features with a Pearson *r* > 0.95 were filtered out, the remaining log-transformed and normalized 453 parameters were used for the *k*-means clustering. The silhouette score showed that the optimal *k* value was four. The resulting *k*-means clusters contained c1 = 213, c2 = 57, c3 = 39 and c4 = 45 segments respectively (Fig. 3). We compared the distribution of anatomic segments, scanner type, liver stiffness, and CT density between the four clusters. The average CT density was significantly higher in c4 (mean ± SD = 110HU ± 10.1HU) than in c1 (96.1HU ± 11.3HU; *p* < 0.0001), c2 (90.8HU ± 16.8HU; p < 0.0001) or c3 (93.1HU ± 17.5HU; p < 0.0001); also the density of segments in c2 was significantly lower (*p* < 0.035) than segments in c1. Meanwhile, there was no significant difference in the distribution of the rest of the variables.

### Univariate analysis of the texture parameters

**Fig. 4:**
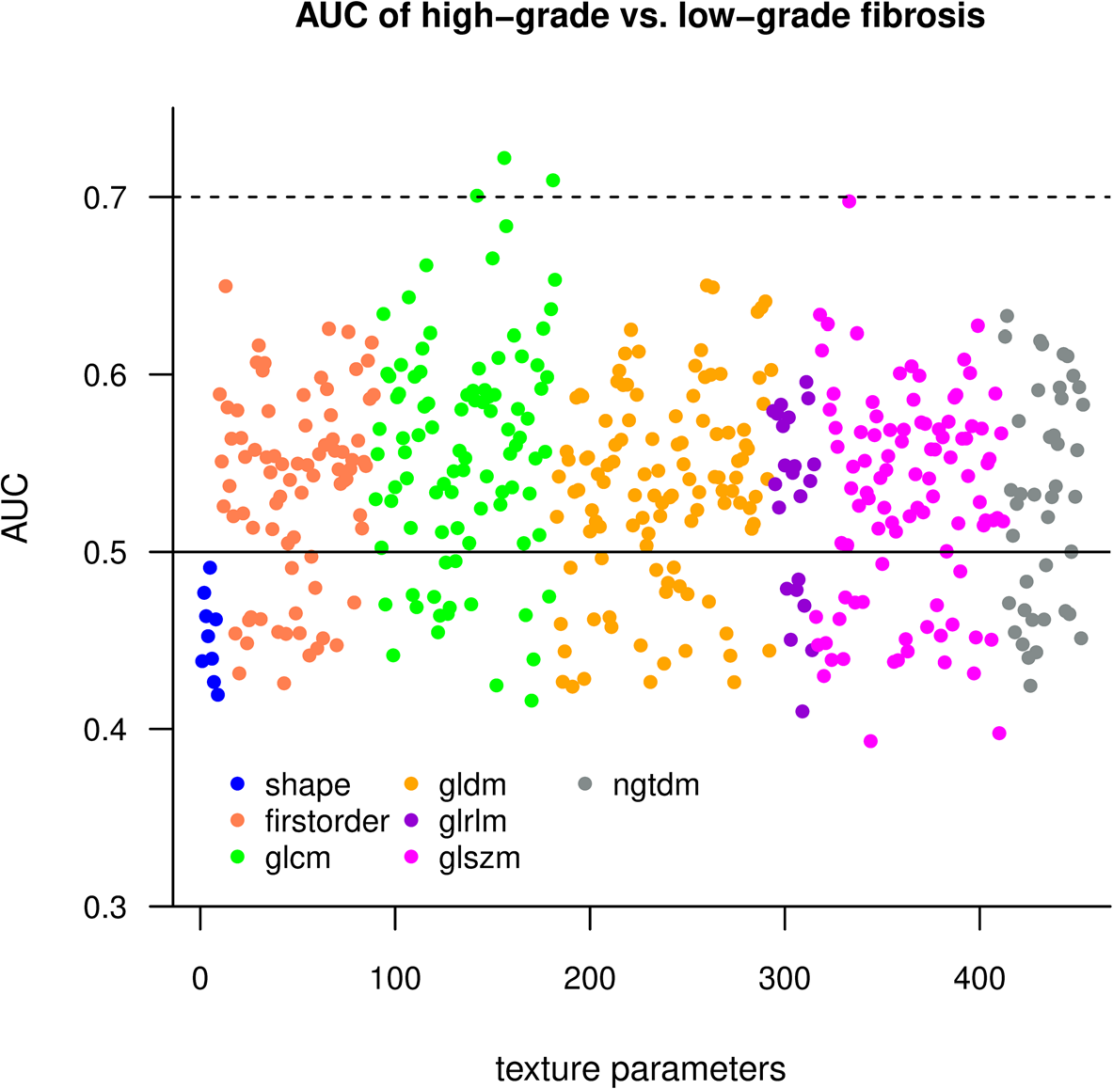
Manhattan plot shows AUC values of different classes of texture features. We calculated the area under the curve (AUC) estimate from 5-fold cross-validation to evaluate individual texture parameters (TP) as classifiers of low-grade vs. high-grade fibrosis. Among the different classes of texture parameters, the features calculated from a grey level co-occurrence matrix (GLCM) had the highest AUC (green dots). Meanwhile, the AUC of the shape-based features (blue dots) did not reach up to the accuracy of other classes. The solid line highlights AUC = 0.5, where features do not have discriminatory power, the best classifiers exceeded AUC = 0.7 (dotted line)

A ROC analysis was performed to test individual texture parameters’ diagnostic ability to differentiate between low-grade and high-grade fibrosis. A cross-validated AUC estimate was calculated to evaluate the individual prediction accuracy of the texture features. A Manhattan plot showed that GLCM features had the highest AUC values, while the shape features performed worse than other metrics (Fig. 4). The top three parameters had a cross-validated AUC > 0.7 and included: wavelet-LLH filtered GLCM Correlation (AUC = 0.72 CI = 0.66–0.78), wavelet-HLH filtered GLCM Informational Measured Correlation (Imc2) (AUC = 0.71 CI = 0.65–0.77) and wavelet-LHL filtered GLCM Correlation (AUC = 0.70 CI = 0.64–0.76). These metrics quantify the complexity of the texture as they describe the correlation between the probability distributions of the GLCM elements.

A univariate linear regression analysis was used to find a relationship between texture parameters and liver stiffness. The R^2^ and *β* coefficients, and the *p*-value were calculated for each parameter from 1000 bootstrap replications. Thirty-eight features showed significant (*p* < 0.0011) association with liver stiffness, although their R^2^ was low. The best predictor of liver stiffness was wavelet-HLH filtered GLCM Imc2 (R^2^ = 0.074, β = − 2.593, *p* < 0.0001). The results of the univariate analysis of the texture features are included in Additional file 3.

### Construction of prediction models for the detection of advanced fibrosis

**Fig. 5:**
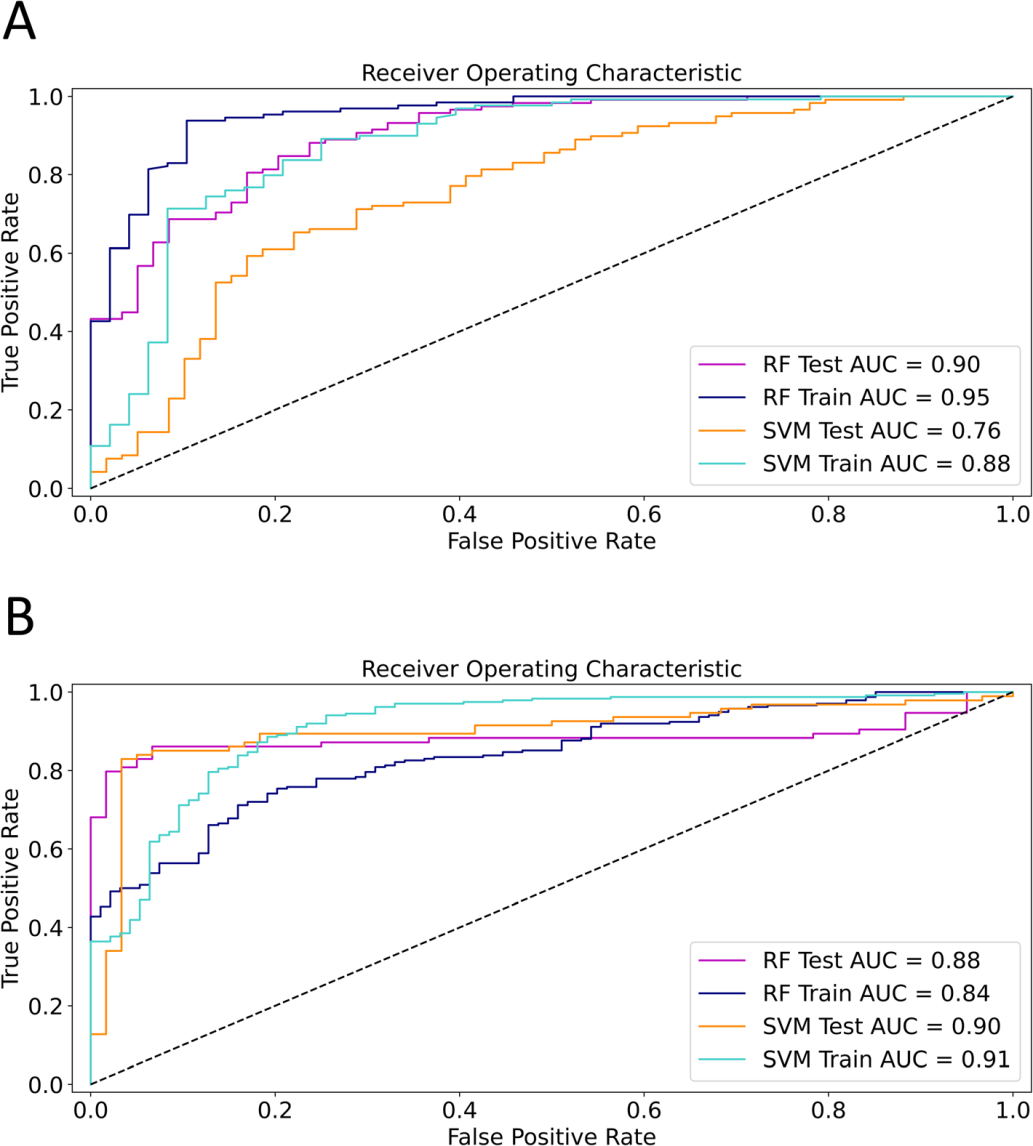
ROC curves of the optimized machine learning models. In the first analysis (**a**) where the liver segments were randomly divided into equal size train and test sets, the random forest classifier (RFC) was able to differentiate between low-grade and high-grade fibrosis with excellent accuracy in the training set (AUC = 0.95, blue line). Its diagnostic ability was only slightly worse in the test set (AUC = 0.90, magenta line). The support vector machine classifier (SVM) achieved very good prediction accuracy in the training set (AUC = 0.88, teal line), and it performed acceptably in the classification of the test set (AUC = 0.76, orange line). In the second analysis (**b**) segments of 64-slice scans were used for training and segments of 16-slice scans for testing the models. The RFC model achieved very good prediction accuracy in both the training (AUC = 0.84, blue line) and test sets (AUC = 0.88, magenta line). The SVM’s accuracy for the prediction of high-grade fibrosis was excellent in both the training (AUC = 0.91, teal line) and the test set (AUC = 0.90, orange line) (**b**)

We trained SVM and RFC machine learning classifiers to differentiate between liver segments with low-grade and high-grade fibrosis based on selected texture features. At first, we randomly split the segments between the train and test sets in a 50:50 ratio. The proportion of cirrhotic and non-cirrhotic liver segments was similar in the training (non-cirrhotic 48, 27%, cirrhotic 129, 73%) and test (non-cirrhotic: 59, 33%, cirrhotic 118, 67%) groups. Next, features with an FDR greater than 10% were removed from the training set. The filtered training set consisted of 177 segments and 154 features. A grid search method was used to fine-tune the SVM and RFC classifiers’ hyperparameters using ten repeats of stratified 5-fold cross-validation of the training set. The less important texture parameters were removed from the models during a cross-validated RFE process. In the first analysis, the optimized RFC model (model 1) included two features (first-order 90Percentile and wavelet-LHH filtered GLCM Inverse-Variance) and achieved an excellent cross-validated classification rate (AUC = 0.95, CI = 0.91–0.98) in the training set **(**Fig. 5A**)**. The optimized SVM model (model 2) included 18 features, and its cross-validated accuracy was very good (AUC = 0.88, CI = 0.81–0.94) for the training set. The diagnostic performance of both models was also evaluated in the test set, where the RFC (model 1) achieved an excellent (AUC = 0.90, CI = 0.85–0.95), and the SVM (model 2) had an acceptable cross-validated accuracy (AUC = 0.76, CI = 0.67–0.84) (Table 2).

**Table 2:** The performance of machine learning classifiers in the prediction of low-grade vs. high-grade fibrosis

Model^**a**^	RFC (Model 1)^**b**^	SVM (Model 2)^**b**^	RFC (Model 3)^**c**^	SVM (Model 4)^**c**^
Number of features^d^	2	18	28	66
Train AUC	0.95 (0.91–0.98)	0.88 (0.81–0.94)	0.84 (0.82–0.85)	0.91 (0.88–0.94)
Test AUC	0.90 (0.85–0.95)	0.76 (0.67–0.84)	0.88 (0.84–0.91)	0.90 (0.87–0.93)
Sensitivity^e^	86%	93%	86%	83%
Specificity^e^	78%	31%	92%	95%
NPV^e^	89%	73%	81%	78%
PPV ^e^	73%	69%	94%	96%

^a^ Optimized for the classification of low-grade vs. high-grade fibrosis in liver segments;

^b^ The liver segments were randomly divided into equal size training and test set;

^c^ Segments of patients who had been scanned with a 64-slice scanner constituted the training set, and patients who had been scanned with a 16-slice scanner were assigned to the test set;

^d^After cross-validated recursive feature elimination;

^e^ Calculated in the test set; *RFC* random forest classifier, *SVM* support vector machine classifier, *NPV* negative predictive value, *PPV* positive predictive value

We also predicted fibrosis in the whole liver (WL) and right lobe (RL) in each patient using the same models, as these segments best correspond to the site of SWE measurements. When these two segments were predicted with the pre-trained machine learning models, the cross-validated accuracy of the RFC (model 1) was better in the right lobe (AUC = 0.81, CI = 0.65–0.98) than in the whole liver (AUC = 0.70, CI = 0.52–0.88). Meanwhile, the SVM (model 2) had lower, but similar prediction accuracy in both right lobe (AUC = 0.67, CI = 0.56–0.78) and the whole liver (AUC = 0.70, CI = 0.64–0.75) segments.

In the second analysis, the liver segments were split based on the type of CT scanner: the segments of patients who had been scanned with a 64-slice scanner constituted the training set (177 segments of 15 patients), and patients who had been scanned with a 16-slice scanner (154 segments of 13 patients) were assigned to the test set (Fig. 2). Two patients scanned with another type of scanner were excluded from the analysis. Thus, we have avoided splitting segments belonging to the same patient between the train and validation sets. The RFC model (model 3) included 28 highest scoring features after RFE and had very good prediction accuracy for both the training (AUC = 0.84, CI = 0.82–0.85) and the test (AUC = 0.88, CI = 0.84–0.91) for differentiating high-grade from low-grade fibrosis. Meanwhile, the SVM model (model 4) used 66 highest scoring features, and its classification accuracy was excellent for the same train (AUC = 0.91, CI = 0.88–0.94) and test sets (AUC = 0.90, CI = 0.87–0.93) (Fig. 5B) (Table 2). Similar to the first analysis, the fibrosis in the whole liver and right lobe could be predicted with both the RFC (model 3) (WL AUC = 0.85, CI = 0.77–0.92, RL AUC = 0.83, CI = 0.79–0.86) and the SVM (model 4) (WL AUC = 0.83, CI = 0.77–0.88, RL AUC = 0.85, CI = 0.81–0.89) with very good accuracy.

There was considerable overlap in the relevant features selected after RFE among the four prediction models. We found that 27 relevant features were selected for at least two, and seven out of these features for three models (Additional file 3).

## Discussion

The timely diagnosis of liver fibrosis is crucial as many times, the progressive course of chronic liver disease can lead to life-threatening complications due to cirrhosis, liver failure, and increased risk of liver cancer. The gold standard method for assessing liver fibrosis is a percutaneous liver biopsy. Bedossa et al. studied the variability in the distribution of fibrosis in the liver parenchyma and its impact on the diagnosis and staging of fibrosis with liver biopsy in patients with chronic hepatitis C virus infection. They stressed that sampling variability caused by the heterogeneity of liver fibrosis is limiting accurate assessment [16]. The potential complications and the cost of liver biopsy can be significant. According to recent guidelines, non-invasive markers, including elastography, are recommended for the initial assessment of liver fibrosis in both HBV and HCV associated liver disease. In contrast, a liver biopsy is only preferred in selected cases where there is uncertainty or potential additional etiologies [1–3]. The American Association for the Study of Liver Diseases encourages the development of new effective non-invasive alternatives to liver biopsy [17].

Image analysis techniques are gaining popularity for the non-invasive detection of liver fibrosis, as these can be retrospectively applied to CT scans and do not require unique instrumentation. A handful of studies have examined the value of CTTA, and their results indicated that the performance of CTTA to discriminate between stages of fibrosis is comparable to other non-invasive techniques such as elastography [9, 8, 6, 7, 18]. Our study is based on a direct comparison of SWE and CTTA. The diagnostic performance of SWE has been extensively evaluated against liver biopsy in multiple etiologies of liver fibrosis. The results showed that liver stiffness values correlated with the histological fibrosis stage, and SWE was an accurate technique for the assessment of significant fibrosis and cirrhosis [19, 10]. Although the SWE evaluates stiffness, a physical property of the parenchyma, while CTTA quantifies the architectural distortion, we found that CTTA can reproduce the fibrosis stage determined by SWE with good accuracy.

The attenuation of liver segments that is predominantly dependent on contrast enhancement was significantly different between the four clusters identified by *k-*means clustering. Thus, individual variations in the dynamic of the PV enhancement can significantly influence TPs. The contrast enhancement irregularities in part can be attributed to the architectural heterogeneity and the disrupted blood supply due to excess tissue deposition in the parenchyma.

Previous reports found that CTTA in PV is superior to arterial and non-enhanced scans but most effective in the equilibrium phase for detecting cirrhosis (8). However, equilibrium phase scans are less frequently performed during abdominal CT; thus, it is not an optimal technique to screen for liver fibrosis. Previous studies uniformly analyzed the liver texture in the PV and proved that CTTA based classification of fibrosis is feasible [6–9, 18, 20]. Similarly, we performed CTTA on the PV series and found that CTTA has very good discriminatory power for advanced fibrosis.

We performed three-dimensional CTTA of the liver volume, this approach, compared to previously described slice-by slice-based analysis, can detect additional details of architectural distortion, and identify novel fibrosis-associated features. We identified wavelet-LLH filtered GLCM Correlation, wavelet-HLH filtered GLCM Imc2, and wavelet-LHL filtered GLCM Correlation among the best predictors of fibrosis stage. GLCM Correlation showed a positive association with increased liver stiffness. These metrics describe the correlation between a pixel and it’s neighbor over the whole image. Therefore, our findings may suggest that a repeating texture pattern, such as cirrhotic nodularity, is detected. Meanwhile, similar to prior reports [8, 6, 7, 18], we found that histogram-based metrics such as wavelet-LHL filtered first-order Median, original first-order 10Percentile and original first-order 90Percentile are highly useful for the correct classification of low-grade vs. high-grade fibrosis. All of these features earned a high importance score during RFE. According to Lubner et al. [7, 18], they found that grey level intensity and entropy are good predictors of fibrotic changes, the strong association between fibrosis stage and pixel intensity-related parameters can be explained by the expanded extracellular compartment and collagen deposition during cirrhosis, which may result in increased attenuation and tissue heterogeneity.

Although different types of CT scanners and reconstruction algorithms were used in our patient cohort, we could complete a successful analysis of the data set by normalization and filtering of the texture parameters. Prior studies have demonstrated that differences in CT reconstruction algorithms have a limited effect on texture parameters compared to other texture analysis parameters such as binning [21]. Previous reports, which evaluated texture parameters in different stages of liver fibrosis, achieved similar classification accuracy with multiple scanners [18]. In their study, Pickhardt et al. demonstrated that CTTA of the liver could be applied retrospectively to routine scans performed with either 16- or 64-slice CT scanners, which may have been obtained for other indications [20].

We tested two types of data analysis strategies, which resulted in a similar classification accuracy of patients with advanced fibrosis. Based on the visual evaluation of unsupervised cluster analysis, we concluded that there was no bias from technical factors, which would universally affect texture parameters and prevent the correct classification of the liver segments. Thus, in the first analysis, each liver segment was considered an independent sample and randomly split between train and test set for the machine learning analysis. RFE proved to be a useful technique to define a subset of best-performing features. It constructed highly efficient models where only a handful of parameters could predict advanced fibrosis with good to excellent accuracy. The optimized RFC model consisted of only two features achieved an excellent diagnostic accuracy in both the train and test sets. The specificity and negative predictive value of the model were also very good, as they reached 86 and 89%, respectively. However, we observed a drop in classification rates between the train and test sets, AUC 0.95 to 0.9, and AUC 0.88 to 0.76 in the case of both RFC and SVM models, respectively. This can be a sign of overfitting of the model on the training set, which can be in part caused by sharing segments from the same patients between the train and test sets. The overfitting was observed even when 10 times repeated stratified 5-fold cross-validation was used during all steps of model building, which is a universally accepted technique in machine learning [22]. Therefore, during a second analysis, segments were divided into train and test sets according to the scanner types. Thus, we could prevent potential overfitting of models by splitting segments belonging to the same patient between the train and test groups. This time, the classification rate did not drop in the test set with either model, which indicated that the models’ overfitting could be reduced.

The diagnostic accuracy of all four models is comparable, or in the case of the model 1, 2 and 3 (90, 88, and 90%, respectively) exceeds the performance of multivariate models described by other authors in previous studies. Kayaalti et al. used CTTA for pairwise comparisons between consecutive stages of fibrosis, and reported 94% mean classification accuracy with an SVM [9]. Another study by Zhang et al., a radial SVM, consisted of 15 texture features and was able to classify cirrhosis and non-cirrhosis with a 66.83% accuracy rate [8].

We also tested the models’ performance for the prediction of fibrosis in individual patients. In the first analysis, the RFC model (model 1) achieved the highest accuracy in the right lobe segments (AUC = 0.81). Meanwhile, in the second analysis, the RFC (model 3) and SVM (model 4) classifiers performed equally well for the prediction of the fibrosis status of the whole liver (AUC = 0.85 vs. AUC = 0.83, respectively) and the right lobe (AUC = 0.83 vs. AUC = 0.85, respectively). These results are comparable with the classification rates achieved with similar, previously published models. The model proposed by Lubner et al. could detect advanced fibrosis (≥F3) with an AUC of 0.82 [18]. Based on the analysis, we advise that one should apply either model 3 to the right lobe or model 4 to the whole liver to evaluate fibrosis in individual patients. Nevertheless, we emphasize that the models’ accuracy in predicting a single segment can be improved if the training is conducted on the dataset consisting of the same segments only.

Our research has several limitations. It is a retrospective study, which was completed in a single institution. The study population included a small patient cohort of 30 cases. However, we analyzed the anatomic liver segments separately, and CTTA was performed on 354 segments. There were mixed etiologies that resulted in chronic liver disease. Therefore, the population investigated was not homogenous. Recent guidelines recommend non-invasive methods for the initial assessment of liver fibrosis in both HBV and HCV associated liver disease. In contrast, liver biopsy is no longer considered as the first-line method in routine daily practice and only preferred in selected cases where there is uncertainty or potential additional etiologies [1–3]. SWE has been extensively validated and recognized as an accurate technique for staging liver fibrosis [10, 23]. Thus, similar to other studies, we used ultrasound elastography for the assessment of advanced-stage fibrosis instead of liver biopsy [24, 25]. Imaging in PV is routinely performed during most of the abdominal CT scans. Thus, fibrosis associated CTTA features identified in PV can be conveniently used for follow-up retrospective assessment and screening for chronic liver diseases.

## Conclusions

This report is among the first to investigate the feasibility of three-dimensional CTTA in liver fibrosis. The results reported in this study clearly demonstrate the value of volume-based CTTA in the evaluation of chronic liver disease by identifying patients who are at risk of complications. We demonstrated that features, which describe grey-level intensities and image heterogeneity, are strongly associated with the progression of fibrosis. Finally, we prove that cross-validated machine learning models based on only a handful of selected features can reliably differentiate between low-grade and high-grade fibrosis on different types of scanners.

## Supplementary information

**Additional file 1.** List of liver segments. The characteristics of 354 anatomic liver segments, including patient ID, anatomic locations, liver stiffness measured with pSWE, attenuation, and type of the CT scanner.

**Additional file 2.** The CTTA dataset with all texture parameters. Details of 1117 texture parameters, including feature ID, class, name, and filter type. Unfiltered values of 1117 texture parameters of 354 anatomic liver segments sorted by feature ID.

**Additional file 3.** Univariate evaluation of the radiomic features. The table contains results of the ROC analysis of texture parameters for the classification of low-grade vs. high-grade fibrosis sorted by the AUC value; the coefficient of a univariate linear regression analysis performed against liver stiffness as the dependent variable, and feature importance scores calculated during cross-validated recursive feature elimination.

## Data Availability

The datasets supporting the conclusions of this article are included within the article and its additional files.

## Abbreviations

AIH: Autoimmune hepatitis
AUC: Area under the receiver-operating characteristics curve
CI: Confidence interval
CTTA: CT texture analysis
FDR: False discovery rate
GLCM: Grey level co-occurrence matrix
GLDM: Grey level dependence matrix
GLRLM: Grey level run-length matrix
GLSZM: Grey level size zone matrix
HBV: Hepatitis B virus
HCV: Hepatitis C virus
HU: Hounsfield unit
Imc: Informational measured correlation
LoG: Laplacian of Gaussian filter
NGTDM: Neighboring grey-tone difference matrix
NPV: Negative predictive value
PBC: Primary biliary cholangitis
PPV: Positive predictive value
PSC: Primary sclerosing cholangitis
pSWE: Point shear-wave elastography
PV: Portal venous phase
RFC: Random forest classifier
RFE: Recursive feature elimination
RL: Right lobe of the liver
ROC: Receiver-operating characteristics
SD: Standard deviation
SVM: Support vector machine classifier
TP: Texture parameter
WL: whole liver

## References

[1] Dietrich CF, Bamber J, Berzigotti A, Bota S, Cantisani V, Castera L (2017). EFSUMB guidelines and recommendations on the clinical use of liver ultrasound Elastography, update 2017 (long version). Ultraschall Med.

[2] European Association for Study of Liver, Higado ALpeEd (2015). EASL-ALEH clinical practice guidelines: non-invasive tests for evaluation of liver disease severity and prognosis. J Hepatol.

[3] Ferraioli G, Filice C, Castera L, Choi BI, Sporea I, Wilson SR (2015). WFUMB guidelines and recommendations for clinical use of ultrasound elastography: part 3: liver. Ultrasound Med Biol.

[4] Muthupillai R, Lomas DJ, Rossman PJ, Greenleaf JF, Manduca A, Ehman RL (1995). Magnetic resonance elastography by direct visualization of propagating acoustic strain waves. Science..

[5] Lubner MG, Smith AD, Sandrasegaran K, Sahani DV, Pickhardt PJ (2017). CT texture analysis: definitions, applications, biologic correlates, and challenges. Radiographics..

[6] Daginawala N, Li B, Buch K, Yu H, Tischler B, Qureshi MM (2016). Using texture analyses of contrast enhanced CT to assess hepatic fibrosis. Eur J Radiol.

[7] Lubner MG, Malecki K, Kloke J, Ganeshan B, Pickhardt PJ (2017). Texture analysis of the liver at MDCT for assessing hepatic fibrosis. Abdom Radiol (NY).

[8] Zhang X, Gao X, Liu BJ, Ma K, Yan W, Liling L (2015). Effective staging of fibrosis by the selected texture features of liver: which one is better, CT or MR imaging?. Comput Med Imaging Graph.

[9] Kayaalti O, Aksebzeci B, Ökkeş Karahan İ, Deniz K, Öztürk M, Yılmaz B (2014). Liver fibrosis staging using CT image texture analysis and soft computing. Appl Soft Comput.

[10] Kaposi PN, Unger Z, Fejer B, Kucsa A, Toth A, Folhoffer A (2020). Interobserver agreement and diagnostic accuracy of shearwave elastography for the staging of hepatitis C virus-associated liver fibrosis. J Clin Ultrasound.

[11] Fedorov A, Beichel R, Kalpathy-Cramer J, Finet J, Fillion-Robin JC, Pujol S (2012). 3D slicer as an image computing platform for the quantitative imaging network. Magn Reson Imaging.

[12] van Griethuysen JJM, Fedorov A, Parmar C, Hosny A, Aucoin N, Narayan V (2017). Computational Radiomics system to decode the radiographic phenotype. Cancer Res.

[13] Dong W, Ren J, Zhang D (2011). Hierarchical K-Means Clustering Algorithm Based on Silhouette and Entropy.

[14] LeDell E, Petersen M, van der Laan M (2015). Computationally efficient confidence intervals for cross-validated area under the ROC curve estimates. Electron J Stat.

[15] Dray S (2008). On the number of principal components: a test of dimensionality based on measurements of similarity between matrices. Comput Stat Data Anal.

[16] Bedossa P, Dargère D, Paradis V (2003). Sampling variability of liver fibrosis in chronic hepatitis C. Hepatology..

[17] Rockey DC, Caldwell SH, Goodman ZD, Nelson RC, Smith AD (2009). Diseases AAftSoL. Liver biopsy Hepatology.

[18] Lubner MG, Jones D, Kloke J, Said A, Pickhardt PJ. CT texture analysis of the liver for assessing hepatic fibrosis in patients with hepatitis C virus. Br J Radiol. 2019;92(1093):20180153. 10.1259/bjr.20180153 PMC6435049 30182750

[19] Ferraioli G, Tinelli C, Lissandrin R, Zicchetti M, Dal Bello B, Filice G (2014). Point shear wave elastography method for assessing liver stiffness. World J Gastroenterol.

[20] Pickhardt PJ, Graffy PM, Said A, Jones D, Welsh B, Zea R (2019). Multiparametric CT for noninvasive staging of hepatitis C virus-related liver fibrosis: correlation with the Histopathologic fibrosis score. AJR Am J Roentgenol.

[21] Kolossvary M, Szilveszter B, Karady J, Drobni ZD, Merkely B, Maurovich-Horvat P (2019). Effect of image reconstruction algorithms on volumetric and radiomic parameters of coronary plaques. J Cardiovasc Comput Tomogr.

[22] Krstajic D, Buturovic LJ, Leahy DE, Thomas S (2014). Cross-validation pitfalls when selecting and assessing regression and classification models. J Cheminform.

[23] Fu J, Wu B, Wu H, Lin F, Deng W (2020). Accuracy of real-time shear wave elastography in staging hepatic fibrosis: a meta-analysis. BMC Med Imaging.

[24] Papadopoulos N, Vasileiadi S, Papavdi M, Sveroni E, Antonakaki P, Dellaporta E (2019). Liver fibrosis staging with combination of APRI and FIB-4 scoring systems in chronic hepatitis C as an alternative to transient elastography. Ann Gastroenterol.

[25] Yamamura S, Kawaguchi T, Nakano D, Tomiyasu Y, Yoshinaga S, Doi Y (2020). Profiles of advanced hepatic fibrosis evaluated by FIB-4 index and shear wave elastography in health checkup examinees. Hepatol Res.

